# Variation in central venous oxygen saturation to evaluate fluid responsiveness: a systematic review and meta-analysis

**DOI:** 10.1186/s13054-023-04480-z

**Published:** 2023-05-26

**Authors:** Jianneng Pan, Yuxiang Sun, Zhaojun Xu, Pingping Dong, Xiaoyang Zhou

**Affiliations:** 1 Department of Intensive Care Medicine, Ningbo No.2 Hospital, Ningbo, 315000 Zhejiang China; 2 Department of Emergency, Ningbo Yinzhou No.2 Hospital, Ningbo, 315000 Zhejiang China; 3 Baihe Street Community Health Services of Yinzhou District, Ningbo, 315000 Zhejiang China

**Keywords:** Central venous oxygen saturation, Cardiac output, Fluid responsiveness, Volume expansion

## Abstract

**Background:**

Since oxygen content and oxygen consumption typically remain unchanged within a short period, variation in central venous oxygen saturation (ΔScvO_2_) during fluid challenge can theoretically track the changes in cardiac output (CO). We conducted this meta-analysis to systematically assess the diagnostic performance of ΔScvO_2_ during a fluid challenge for fluid responsiveness in mechanically ventilated patients receiving volume expansion.

**Methods:**

Electronic databases were systematically searched to identify relevant studies published before October 24, 2022. As the cutoff value of ΔScvO_2_ was expected to vary across the included studies, we estimated the area under the hierarchical summary receiver operating characteristic curve (AUHSROC) as the primary measure of diagnostic accuracy. The optimal threshold of ΔScvO_2_ and the corresponding 95% confidential interval (CI) were also estimated.

**Results:**

This meta-analysis included 5 observational studies comprising 240 participants, of whom 133 (55%) were fluid responders. Overall, the ΔScvO_2_ during the fluid challenge exhibited excellent performance for defining fluid responsiveness in mechanically ventilated patients receiving volume expansion, with an AUHSROC of 0.86 (95% CI 0.83–0.89), a pooled sensitivity of 0.78 (95% CI 0.69–0.85), a pooled specificity of 0.84 (95% CI 0.72–0.91), and a pooled diagnostic odds ratio of 17.7 (95% CI 5.9–53.2). The distribution of the cutoff values was nearly conically symmetrical and concentered between 3 and 5%; the mean and median cutoff values were 4% (95% CI 3–5%) and 4% (95% CI not estimable), respectively.

**Conclusions:**

In mechanically ventilated patients receiving volume expansion, the ΔScvO2 during the fluid challenge is a reliable indicator of fluid responsiveness.

*Clinical trial registration* PROSPERO, https://www.crd.york.ac.uk/prospero/, registry number: CRD42022370192.

**Supplementary Information:**

The online version contains supplementary material available at 10.1186/s13054-023-04480-z.

## Background

Volume expansion is one of the first-line treatments of hypotension and hypoperfusion in critically ill patients. Fluid administration is anticipated to increase cardiac output (CO) and oxygen delivery (DO_2_), and finally restore tissue perfusion, while accompanied by a high risk of fluid overloading if cardiac performance has reached the plateau of the Frank–Starling curve. Thus, the assessment of fluid responsiveness is a crucial procedure before fluid infusion. Seeking reliable surrogate indicators of fluid responsiveness has always been an important research issue in critical care medicine. As fluid responsiveness is usually defined by a significant increase (≥ 15%) in CO after receiving a certain amount of fluid within a short period [1, 2], parameters associated with the changes of CO or CO-derived indices, theoretically, possess the potential to be surrogate markers of fluid responsiveness.

Given the close relationship between blood flow (i.e., CO) and energy metabolism, parameters related to oxygen metabolism or carbon dioxide production are widely studied to define fluid responsiveness [3–6]. Mixed venous oxygen saturation (SvO_2_), which is clinically substituted by central venous oxygen saturation (ScvO_2_) in adequate circumstances due to the equivalent change trend of the two variables [7, 8], has historically been considered as an oxygen metabolic variable that may reflect the balance between DO_2_ and oxygen consumption (VO_2_). Since VO_2_ typically remains unchanged during a fluid challenge, the variation in ScvO_2_ (ΔScvO_2_) during the fluid challenge can theoretically track the change of DO_2_, which was confirmed in human and animal subjects with different cardiovascular conditions [9, 10]. As a consequence, the ΔScvO_2_ during the fluid challenge may also reflect the change of CO because oxygen content is, at least theoretically, constant within a short time. These rationales underlie the ability of ΔScvO_2_ to evaluate fluid responsiveness. Currently, the diagnostic performance of ΔScvO_2_ for fluid responsiveness remains inconclusive, even though the relationship between the changes of ScvO_2_ and CO during fluid challenge has been widely studied [3–5]. Therefore, we conducted this meta-analysis to systematically assess the diagnostic accuracy of ΔScvO_2_ for evaluating fluid responsiveness.

## Method

This meta-analysis was reported following the Preferred Reporting Items for a Systematic Review and Meta-analysis of Diagnostic Test Accuracy [11]. We registered the study protocol at the international prospective register of systematic reviews (PROSPERO, CRD42022370192) before initiating the study.

### Data sources and search strategy

Systematic literature searching was conducted by two independent reviewers (JP and YS) on October 24, 2022, in four electronic databases (PubMed, Web of Science, Embase, and Cochrane Library) to retrieve studies that assessed the diagnostic accuracy of the ΔScvO_2_ during the fluid challenge for fluid responsiveness in mechanically ventilated patients receiving volume expansion, without any date or language restriction. The bibliographies of relevant publications were reviewed to further identify relevant articles. The detailed search strategies are presented in Additional file 1: Table S1.

### Eligibility criteria

Candidate studies were screened in compliance with the following eligibility criteria: (1) conducted on mechanically ventilated adults (age > 18 years), whose physicians in charge decided to administrate fluid to restore tissue hypoperfusion or hypotension; (2) assessing fluid responsiveness (the reference index) in the context of a fluid challenge; (3) measuring ScvO_2_ (the index test) before and immediately after fluid challenge, and calculating ΔScvO_2_ by the relative changes of ScvO_2_ from the baseline value; and (4) reporting sufficient information to construct a 2 × 2 contingency table. No restriction was applied to the definition of fluid responsiveness. We excluded studies that met anyone of the following criteria: (1) volume expansion was not conducted in the form of fluid challenge, that is, the volume of fluid was more than 500 mL or administrated for more than 30 min; (2) measuring SvO_2_ instead of ScvO_2_; or (3) conference abstracts without a full text.

### Study selection, data extraction, and quality assessment

Initially, two independent reviewers (ZX and PD) checked all searched records for duplicates and performed deduplication. After that, they reviewed the title and abstract of the remaining records for relevance. Finally, the full text of candidate studies was carefully reviewed to determine their suitability for inclusion or exclusion. Any disagreement between the two reviewers was resolved through discussion with a third reviewer (XZ).

The same two reviewers (ZX and PD) pre-customized an extraction form to extract the study and patient characteristics from each included study. They also recorded the diagnostic accuracy of ScvO_2_ for fluid responsiveness in detail in Additional file 1: Table S2, including the area under the receiver operating characteristic (ROC) curve (AUROC), the sensitivity, the specificity, and its corresponding cutoff value. According to the diagnostic accuracy (sensitivity and specificity) and sample size in each included study, we calculated the true positive, false positive, false negative, and true negative values to construct a 2 × 2 contingency table. The corresponding authors would be contacted to inquire about the missing data if necessary. A joint review of articles was suggested to resolve any disagreements between the two reviewers.

The methodological quality of each included study was independently assessed by two reviewers (YS and XZ) using the Quality Assessment of Diagnostic Accuracy Studies (QUADAS)-2 tool [12]. If existing disagreements, a discussion would be required to achieve a consensus.

### Statistical analysis

Due to the largely varied patient characteristics, we expected substantial between-study variations between the included studies. Therefore, before data synthesis, the derived estimates of sensitivity and specificity from each included study were plotted on forest plot and ROC space to explore the between-study variations in the diagnostic accuracy of ΔScvO_2_ for fluid responsiveness. We adopted the random-effect bivariate model to calculate the pooled sensitivity, pooled specificity, and pooled diagnostic odds ratio (DOR) [13, 14], where the bivariate model integrates a correlation parameter allowing for the expected trade-off in sensitivity and specificity due to the varied threshold values of ΔScvO_2_ across the included studies [13]. The DOR is an appropriate global measure of comparing the accuracy of different diagnostic tests, and it can take values between 0 and infinity. The higher the DOR value, the better the diagnostic performance. A value equal to 1 indicates that a test does not discriminate between patients with and those without the target condition [15]. However, we reported the area under the hierarchical summary ROC (HSROC) curve (AUHSROC), where the HSROC curve was fitted using the HSROC model [16], as the primary measure of diagnostic accuracy because of the expected threshold effect [13]. The pooled sensitivity and specificity cannot be reasonably suggested as main measures of diagnostic accuracy because they represent estimates of a certain notional unspecified average of different thresholds that cannot be clinically interpreted [13]. Consequently, we estimated the optimal threshold value of ΔScvO_2_ by calculating the mean and median values, while observing the distribution, dispersion, and central tendency of the cut-off values. Statistical analyses were performed using Stata/SE 15.0 software with the MIDAS and METANDI modules (Stata-Corp, College Station, TX, USA). A two-tailed *P* < 0.05 indicated statistical significance.

We assessed the between-study heterogeneity by using Cochran’s *Q* test and *I*^2^ statistics. The threshold effect was evaluated statistically by calculating the Spearman correlation coefficient between the logit of sensitivity and the logit of 1-specificity [17]. Meanwhile, a Bayesian nomogram was constructed to calculate the posttest probability to facilitate the interpretation of results. To confirm the stability of the present study, we conducted a sensitivity analysis by excluding the studies introducing a high risk of bias. If more than 10 studies were included, we would assess the publication bias by using Deeks’ funnel plot asymmetry test [18]. Additionally, we would conduct subgroup analyses according to the baseline lactate and ScvO_2_ level if sufficient studies were identified because the two variables may reflect what stage is the VO_2_/DO_2_ dependency at [19, 20].

## Results

**Fig. 1:**
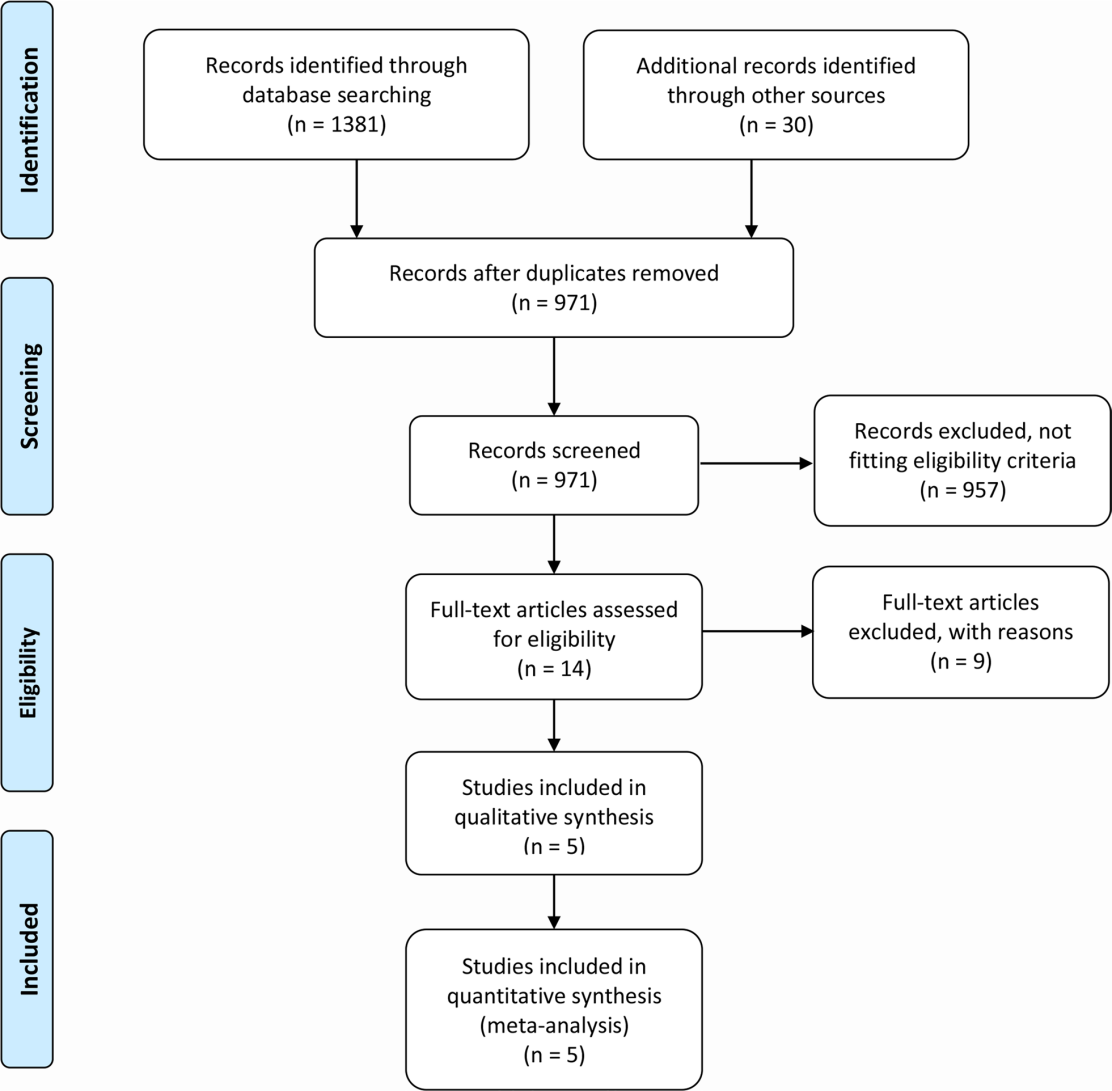
PRISMA flowchart of study selection

We identified a total of 1381 records from electronic database searching and manually selected additional 30 records from other publications. After deduplication and precluding irrelevant records, we carefully reviewed the full text of 14 candidate studies. Finally, five studies [3–5, 21, 22] met the eligibility criteria and were included in the quantitative analysis. Figure 1 depicts the PRISMA flowchart of study selection.

### Baseline characteristics

All included studies were prospective designs and published in the past decade, with a sample size ranging from 30 to 88. Among the included studies, 4 [3, 4, 21, 22] were conducted in the intensive care unit (ICU) setting and 1 [5] was performed in the emergency department. In 3 studies [5, 21, 22], all subjects (100%) were mechanically ventilated at the time of fluid challenge; in the remaining 2 studies [3, 4], most subjects (more than 70%) received mechanical ventilation during the study period. Of note, the definitions of fluid responsiveness and fluid challenge varied across the included studies. CO was measured using the thermodilution method [3–5, 22] or echocardiography [21]. Table 1 records the baseline characteristics of each included study in detail.

**Table 1:** Baseline characteristics of the included studies

Study No.	Author/year	Design, setting, and location	Subjects	Sample size	Invasive mechanical ventilation (n, %)	Age (mean, years)	Baseline lactate (mean, mmol/L)	Baseline ScvO_2_ (mean, %)	Fluid challenge	Definition of fluid responsiveness	CO measurement method	No. of fluid responder (n, %)
1	Giraud/2011	Prospective study; ICU; Switzerland	Hypotensive patients (MAP < 60 mm Hg) secondary to cardiogenic shock or postoperative cardiac surgery	30	21 (70)	71	NR	63	Saline, 500 mL, within 10 min	Cardiac index increase ≥ 15%	Thermodilution using PAC	14 (47)
2	Xu/2017	Prospective study; ICU; China	Septic shock patients with invasive CO monitoring requiring fluid challenge	40	37 (93)	60	5.1	71	Normal saline or 4% gelatin, 500 mL, within 15 min	Cardiac index increase ≥ 10%	Thermodilution using PAC or PICCO	18 (45)
3	Giraud/2021	Prospective study; ICU; Switzerland	Elective postoperative cardiac surgery patients who mechanically ventilated and needed volume expansion	33	33 (100)	63	1.5	65	Saline, 500 mL, within 10 min	VTI increase ≥ 15%	Pulsed-wave Doppler using echocardiography	15 (45)
4	Khalil/2021	Prospective study; Emergency department; Tunisia	Hemodynamically unstable patients under invasive mechanical ventilation who required volume expansion	88	88 (100)	70	3.6	71	Normal saline, 500 mL, within 10 min	CO increase ≥ 15%	Thermodilution method	61 (69)
5	Nassar/2021	Prospective study; ICU; France	Mechanically ventilated sepsis patients with tissue hypoperfusion requiring volume expansion	49	49 (100)	67	1.9	65	Colloid solution (4% Human serum albumin), 500 mL, over 15 min	Cardiac index increase ≥ 10%	Thermodilution using PICCO	25 (51)

*No.* Number; *MAP* Mean arterial pressure; *CO* Cardiac output; *ICU* Intensive care unit; *NR* No record; *ScvO*_*2*_ Central venous oxygen saturation; *VTI* Velocity time integral; *PAC* Pulmonary artery catheter; *PICCO* Pulse indicator continuous cardiac output

### Quality assessment

In the domains of patient selection and index test, all included studies were judged as unclear risk of bias due to lacking sufficient information to support these judgments. However, the reference standard in two studies [4, 22] might introduce bias because they used a cardiac index increase of ≥ 10% to define fluid responsiveness; this threshold, lower than the general threshold [1], might potentially increase the proportion of fluid responder. Furthermore, the study by Nassar et al. [22] had a high concern regarding applicability for reference standard because the duration of the fluid challenge was over 15 min. Table 2 lists the detailed presentation of methodological quality assessment.

**Table 2:** Methodological quality of each included study

Study (author/year)	Risk of bias				Applicability concerns		
	Patient selection	Index test	Reference standard	Flow and timing	Patient selection	Index test	Reference standard
Giraud/2011	?	?	☺	☺	☺	☺	☺
Xu/2017	?	?	☹	☺	☺	☺	☺
Giraud/2021	?	?	☺	☺	☺	☺	☺
Khalil/2021	?	?	☺	☺	☺	☺	☺
Nassar/2021	?	?	☹	☺	☺	☺	☹

☺ low risk; ☹ high risk; ? unclear risk

### Primary analysis

**Fig. 2:**
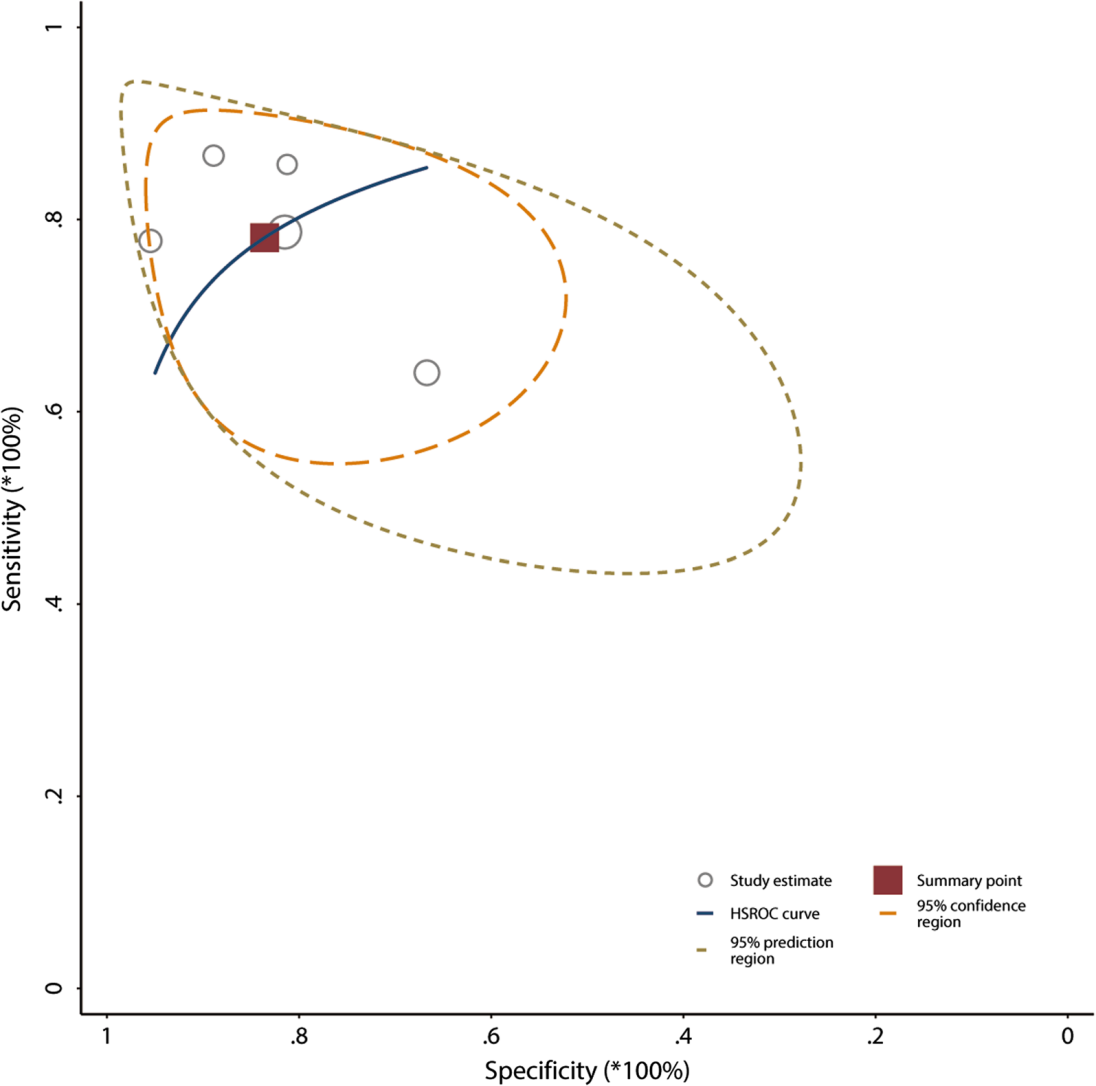
HSROC curve of the ΔScvO_2_ for defining fluid responsiveness. The area under the hierarchical summary receiver operating curve was 0.86 (95% CI 0.83–0.89). The size of the circles indicates the weight of each individual study. The summary point (maroon solid square) represents the average sensitivity and specificity estimates from the study results, and its corresponding 95% confidence region (orange dashed line) is illustrated. The 95% prediction region represents the confidence region for a forecast of true sensitivity and specificity in a future study. HSROC hierarchical summary receiver operating characteristic; ΔScvO_2_ variation in central venous oxygen saturation during the fluid challenge

**Fig. 3:**
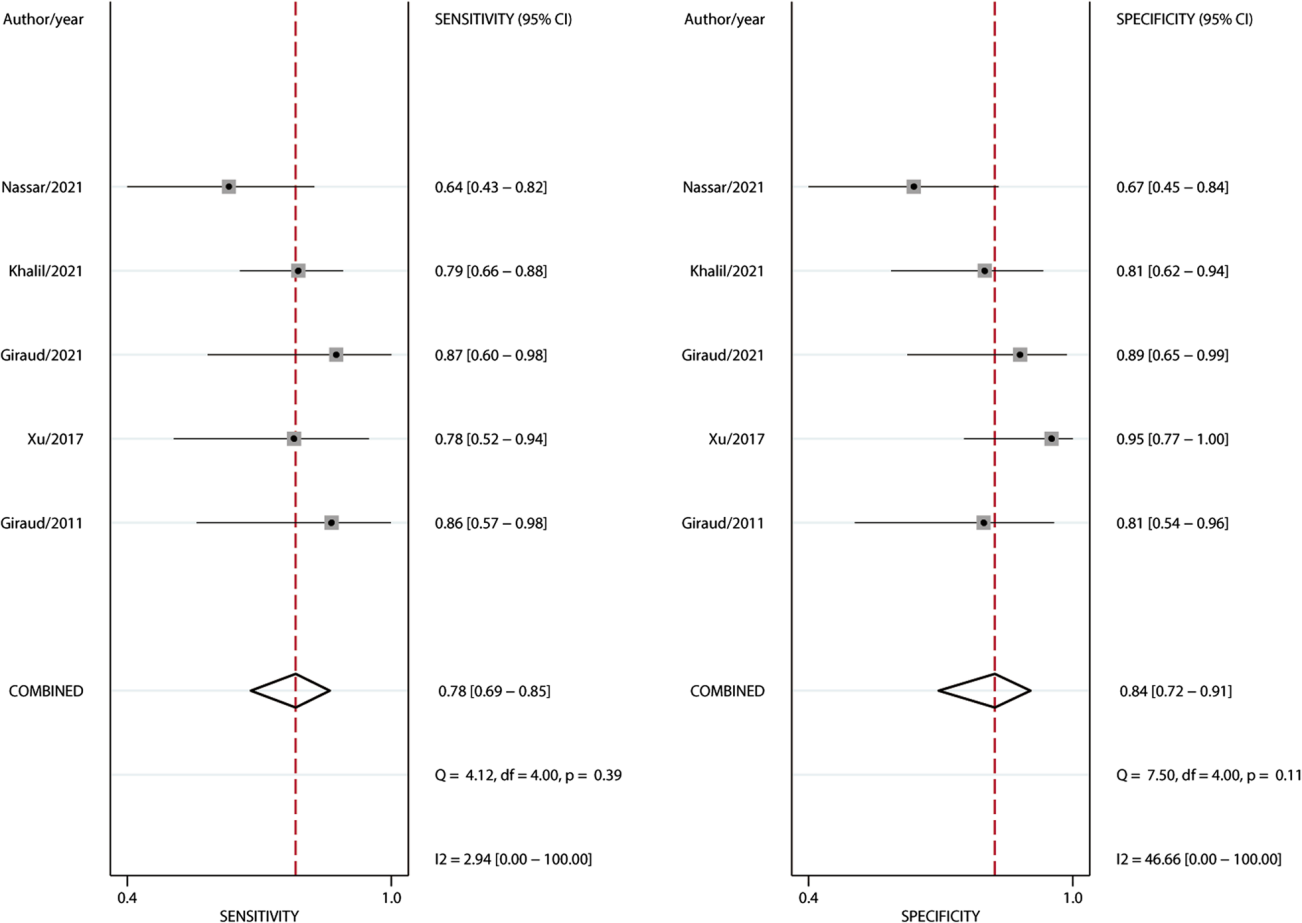
Forest plot of sensitivity and specificity of the ΔScvO_2_ for defining fluid responsiveness. ΔScvO_2_ variation in central venous oxygen saturation during the fluid challenge

**Fig. 4:**
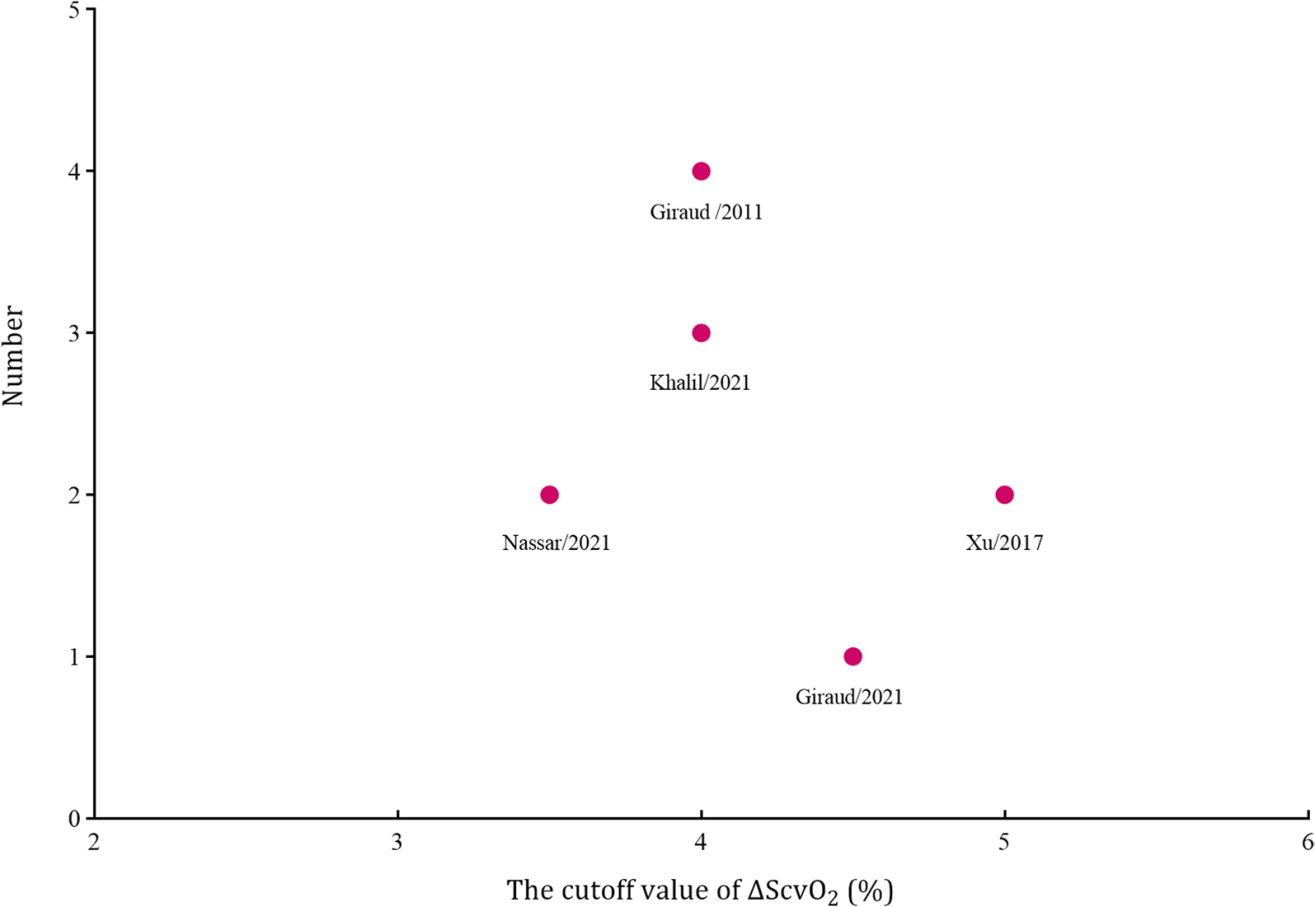
Scatter plot of the cutoff values of the ΔScvO_2_. The distribution of the cutoff values was nearly conically symmetrical and concentered between 3 and 5%. ΔScvO_2_ variation in central venous oxygen saturation during the fluid challenge

**Fig. 5:**
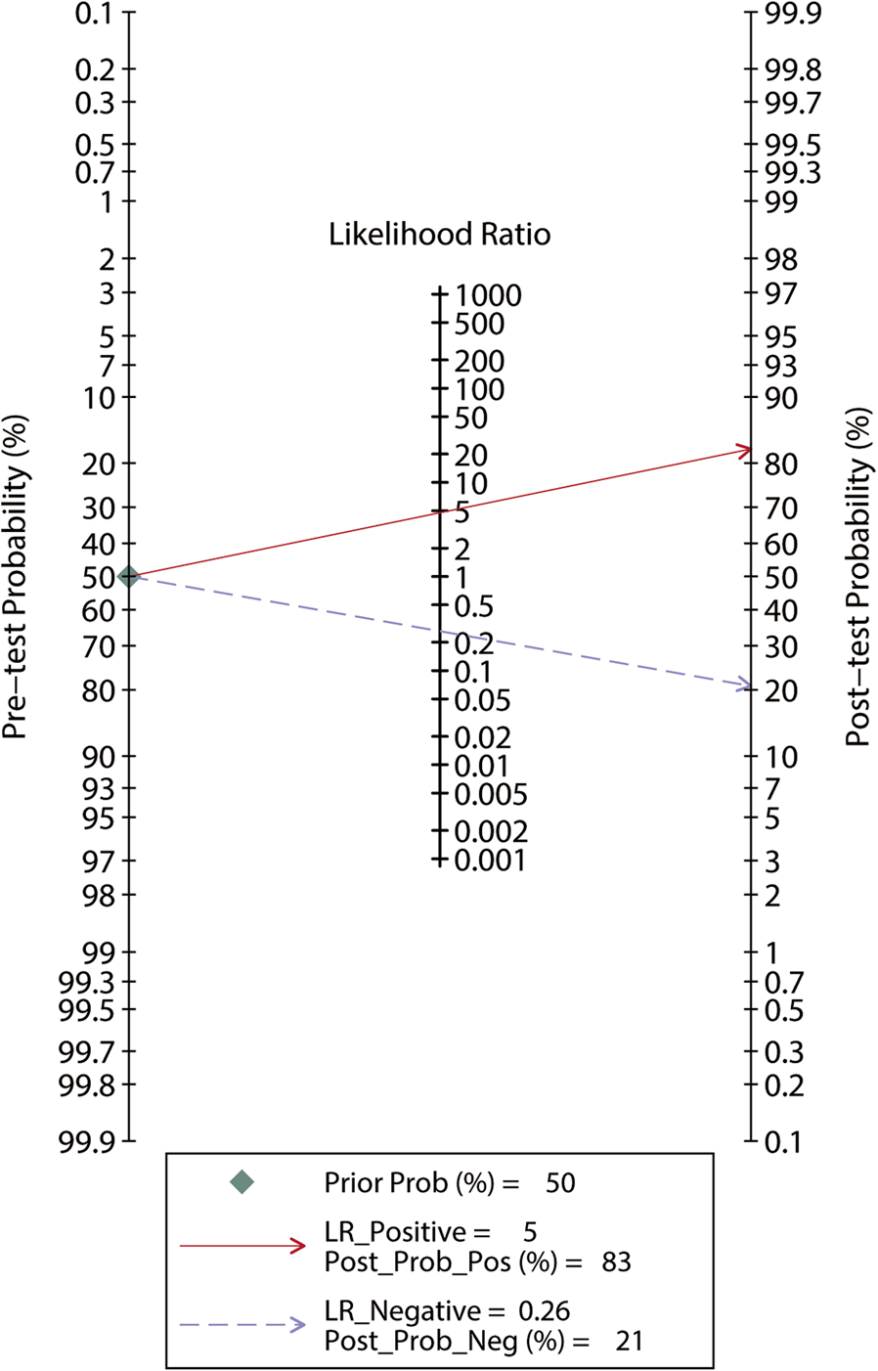
Bayes nomogram of the ΔScvO_2_ for defining fluid responsiveness. If an average-risk population has an assumed pretest probability of fluid responder of 50%, the probability of fluid responder will increase to 83% when the test is positive and decrease to 21% when the test is negative

The 5 included studies enrolled a total of 240 participants, of whom 133 (55%) were fluid responders. All included studies reported the cutoff value of ΔScvO_2_, which ranged from 3.5 to 5%. No between-study heterogeneity was observed, with a Cochran *Q* statistic of 0.315 (*P* = 0.420) and an overall *I*^2^ of 0%. The pooled results suggested that the ΔScvO_2_ during fluid challenge exhibited excellent performance for defining fluid responsiveness, with an AUHSROC of 0.86 [95% confidence interval (CI) 0.83–0.89] (Fig. 2). We also found no significant heterogeneity in sensitivity and specificity (Fig. 3), and the small difference between the 95% confidence and prediction regions consistently indicated no substantial heterogeneity between the studies (Fig. 2). The summarized diagnostic accuracies indicated a pooled sensitivity of 0.78 (95% CI 0.69–0.85), a pooled specificity of 0.84 (95% CI 0.72–0.91), and a pooled DOR of 17.7 (95% CI 5.9–53.2). The scatter plot of the cutoff values of ΔScvO_2_ presented a nearly conically symmetrical distribution (Fig. 4), and the mean and median cutoff values were 4% (95% CI 3–5%) and 4% (95% CI not estimable), respectively. Thus, the range of 3–5% may represent the optimal CI of ΔScvO_2_ for the evaluation of fluid responsiveness. Accordingly, as shown in the Bayes nomogram (Fig. 5), if an average-risk population has an assumed pretest probability of fluid responder of 50% (as estimated by this meta-analysis), the probability of fluid responder will increase to 83% when the ΔScvO_2_ is greater than 5% and decrease to 21% when the ΔScvO_2_ is less than 3%.

As two studies [4, 22] used a cardiac index increase of ≥ 10% to define fluid responsiveness, which introduced a high risk of bias in the domain of reference standard (Table 2), we excluded the two studies to conduct a sensitivity analysis. The sensitivity analysis indicated that the AUHSROC (0.89, 95% CI 0.86–0.92), the pooled sensitivity (0.81, 95% CI 0.72–0.88), and the pooled specificity (0.84, 95% CI 0.72–0.91) (Additional file 1: Fig. S1) were comparable to the primary analysis, confirming the robustness of our results. Given the limited included studies, we did not assess the publication bias, and we also abandoned the scheduled plan of conducting stratified analysis on the baseline lactate level or ScvO_2_ level.

## Discussion

This systematic meta-analysis of 5 observational studies evaluated the diagnostic accuracy of the ΔScvO_2_ during the fluid challenge for fluid responsiveness in mechanically ventilated patients receiving volume expansion. The principal findings indicated that the ΔScvO_2_ during the fluid challenge is a reliable indicator of fluid responsiveness, and the range of 3–5% may represent its optimal CI for evaluating fluid responsiveness.

To the best of our knowledge, this is the first systematic meta-analysis to explore the diagnostic accuracy of ΔScvO_2_ in defining fluid responsiveness. As a potential marker of fluid responsiveness, ΔScvO_2_ has several advantages over conventional variables. First, tracking the changes in ScvO_2_ during a fluid challenge can not only assess the response of CO to a fluid bolus but also evaluate the metabolic efficacy of fluid administration. Furthermore, unlike other dynamic variables associated with heart–lung interactions, ΔScvO_2_ would not, in theory, be affected by arrhythmia, tidal volume, or lung compliance [2, 23]. Therefore, this meta-analysis provides an important clinical implication that ΔScvO_2_ has the potential to become a surrogate marker of fluid responsiveness when CO measurement is unavailable or not applicable. However, before the wide application of ΔScvO_2_ in clinical practice, some prerequisites should be recognized. According to the simplified formula [24], SvO_2_ is estimated as arterial oxygen saturation (SaO_2_)–VO_2_/(1.34 × hemoglobin × CO), where SvO_2_ can be substituted by ScvO_2_ given the good correlation between the two variables [8]. Thus, the changes in ScvO_2_ after volume expansion can theoretically track the fluid-induced changes in CO as long as SaO_2_, VO_2_, and hemoglobin keep unchanged during the fluid challenge. However, the assumption that hemoglobin keeps unchanged during the fluid challenge is not always true. Administering large volumes of fluids within a short period will inevitably result in a decreased hemoglobin concentration. Previous studies have demonstrated that the hemoglobin concentration was decreased by 6–9% after a 500 mL of fluid challenge [4, 19], which was further confirmed by a recent meta-analysis [25]. Accordingly, the potential reduction in hemoglobin concentration might contribute to a decreased ScvO_2_ after volume expansion (i.e., a lower ΔScvO_2_). The negative effect of the reduced hemoglobin concentration might result in an underestimation, not overestimation at least, of the diagnostic performance of ΔScvO_2_ for fluid responsiveness.

Likewise, VO_2_ is not always constant during volume expansion. Numerous studies found the VO_2_/DO_2_ dependency phenomenon in septic patients when DO_2_ was increased through administrating fluid [26–28]. Theoretically, at the VO_2_/DO_2_ dependence stage, VO_2_ and DO_2_ would change linearly, and ScvO_2_ would be unchanged despite the fluid-induced increase in DO_2_ because of the relatively constant oxygen extraction, and thus might not reflect the fluid-induced changes in CO [29]. In other words, ΔScvO_2_ will not be a reliable marker of fluid responsiveness if oxygen metabolism is in the VO_2_/DO_2_ dependency state. However, the above-mentioned theoretical conjecture does not imply the invalidity of ΔScvO_2_ to evaluate the cardiac response to a fluid bolus, because the pathologic DO_2_/VO_2_ dependency phenomenon might be not always common in critically ill patients. For example, in the study by Xu et al. [4], only 2 of 18 included septic shock patients who were fluid responsive exhibited a significant increase in VO_2_ immediately after fluid challenge; other studies [19, 20, 30] recorded a proportion of approximately 50% of septic patients in whom a fluid-induced DO_2_ increase did not lead to a significant increase in VO_2_. In this case, selecting the appropriate cohort to utilize ΔScvO_2_ to define fluid responsiveness would be an interesting issue. Since DO_2_ decreases below the critical value and cannot meet oxygen demand, oxygen debt will be produced at the VO_2_/DO_2_ dependency stage, resulting in an elevated lactate level [19, 29]. In this consideration, we speculate that lactate might help to discriminate against those patients who can use ΔScvO_2_ to define fluid responsiveness and those who cannot. We initially planned to perform subgroup analyses based on the baseline lactate level. However, the scheduled plan of conducting stratified analysis was abandoned due to the limited included studies. Hence, this hypothesis should be verified in future research.

This meta-analysis presents a major methodological strength that the 95% CI of the optimal cutoff value was estimated to avoid the binary constraint of a “black-or-white” decision of the ROC curve and fit the reality of clinical or screening practice. In a realistic clinical decision-making scenario, a single threshold seemly cannot meet the demand of defining fluid responsiveness. For instance, if the measured ΔScvO_2_ is slightly higher or lower than the cutoff value, it is difficult to judge whether the patient will benefit from volume expansion. Hence, we estimated the optimal cutoff value as well as its corresponding CI to overcome this limitation. According to the estimated CI and the Bayes nomogram, we established a feasible decision-making algorithm: (1) if the measured ΔScvO_2_ is greater than 5%, the patient is expected to have a probability of 83% to be a fluid responder; (2) the probability of being fluid responder will decrease to 21% if the measured ΔScvO_2_ is less than 3%; and (3) if the measured ΔScvO_2_ is between 3 and 5%, the fluid responsiveness is difficult to determine; in this case, other methods are suggested to assess fluid responsiveness.

Several limitations in our study should be acknowledged. First, the limited included studies and the relatively small sample sizes (240 patients) represent the primary limitation, resulting in a decreased statistical power that hampered us from drawing a firm conclusion. Furthermore, studies with a small sample size may overestimate the effect sizes. Additionally, several subgroup analyses were not performed due to the limited included studies. For instance, the baseline lactate level and ScvO_2_ level may be associated with the diagnostic performance of ΔScvO_2_; the fluid type (synthetic colloids, albumin, or crystalloids) might cause a potential bias in the results because of the different volume expansion power. Second, all of the included studies had low methodological quality. Of note, two studies [4, 22] might introduce an important bias in our results because they applied a lower threshold value (10%) to define fluid responsiveness, which might increase the proportion of fluid responders. However, after excluding the two studies [4, 22], the sensitivity analysis confirmed the robustness of our findings. Nevertheless, these methodological shortcomings might intrinsically lead to a potential bias in our results and thereby restrict the validity and applicability of our findings. Third, the range of optimal CI (3–5%) is close to the measurement error of ScvO_2_ using a point-of-care blood gas analyzer [31], which could degrade the credibility of our findings to some extent. Thus, it necessitates a standardization of blood gas analysis in clinical practice to reduce measurement error. Lastly, the current findings are only applicable to critically ill patients under mechanical ventilation. It is unclear whether ΔScvO_2_ can evaluate fluid responsiveness in patients with spontaneous breathing. Theoretically, ΔScvO_2_ can also reliably evaluate fluid responsiveness in patients with spontaneous breathing as long as they stay quiet during the fluid challenge so that VO_2_ will not be affected by other factors (such as emotion or activity).

## Conclusion

In mechanically ventilated patients receiving volume expansion, the measurement of ΔScvO_2_ during the fluid challenge is a useful and reliable approach to evaluate fluid responsiveness. The range of 3–5% may represent the optimal confidence interval of ΔScvO_2_ to define fluid responsiveness. Given the low methodological quality of the included studies, larger studies with high methodological quality are warranted to validate the applicability of ΔScvO_2_ in evaluating fluid responsiveness.

## Supplementary Information

**Additional file 1: Table S1.** Detailed searching strategies in each database. **Table S2.** Detailed diagnostic accuracy of ScvO_2_ variation for evaluating fluid responsiveness. **Figure S1.** Sensitivity analysis to assess the robustness of ScvO_2_ variation for defining fluid responsiveness by excluding studies introducing a high risk of bias

## Data Availability

All data generated or analyzed during this study are included in this published article (and its supplementary information files).

## Abbreviations

CO: Cardiac output
DO_2_: Oxygen delivery
VO_2_: Oxygen consumption
ScvO_2_: Central venous oxygen saturation
ΔScvO_2_: Variation in ScvO_2_
SvO_2_: Mixed venous oxygen saturation
ROC: Receiver operating characteristic
AUROC: Area under the ROC curve
HSROC: Hierarchical summary ROC
AUHSROC: Area under the HSROC curve
ICU: Intensive care unit
DOR: Diagnostic odds ratio
CI: Confidence interval
SaO_2_: Arterial oxygen saturation
