# Supplementary material for: Variation in central venous oxygen saturation to evaluate fluid responsiveness: a systematic review and meta-analysis
Source: Crit Care. 2023 May 26;27:203. doi: 10.1186/s13054-023-04480-z (PMC10223907; doi:10.1186/s13054-023-04480-z)
Supplement: Supplementary file 1 — Additional file 1: Table S1. Detailed searching strategies in each database. Table S2. Detailed diagnostic accuracy of ScvO2 variation for evaluating fluid responsiveness. Figure S1. Sensitivity analysis to assess the robustness of ScvO2 variation for defining fluid responsiveness by excluding studies introducing a high risk of bias [file 13054_2023_4480_MOESM1_ESM.docx]

**ADDITIONAL FILE 1.**

Table of Contents

[Table S1. Detailed searching strategies in each database 2](#_Toc132700778)

[Table S2. Detailed diagnostic accuracy of ScvO_2_ variation for evaluating fluid responsiveness 4](#_Toc132700779)

[Figure S1. Sensitivity analysis to assess the robustness of ScvO_2_ variation for defining fluid responsiveness by excluding studies introducing a high risk of bias 5](#_Toc132700780)

## Table S1. Detailed searching strategies in each database

| **PUBMED (355 records)** | | |
| --- | --- | --- |
| **#** | **Details** | **Records** |
| 1 | ((((Vena Cava, Superior[MeSH Terms]) OR (Superior Vena Cavas)) OR (Vena Cavas, Superior)) OR (Superior Vena Cava)) OR (Central venous*) | 64366 |
| 2 | (((((Oxygen saturation[MeSH Terms]) OR (Saturation, Oxygen)) OR (Blood Oxygen Levels)) OR (Blood Oxygen Level*)) OR (Level, Blood Oxygen)) OR (Oxygen Level, Blood) | 102175 |
| 3 | #1 AND #2 | 2275 |
| 4 | (((((ScvO2) OR (SvO2)) OR (Central venous oxygen saturation)) OR (Central venous blood oxygen saturation)) OR (Mixed venous oxygen saturation)) OR (Mixed venous blood oxygen saturation) | 3726 |
| 5 | #3 OR #4 | 4378 |
| 6 | ((((((((Fluid responsiveness) OR (Volume responsiveness)) OR (Preload responsiveness)) OR (Fluid challenge)) OR (Volume challenge)) OR (Fluid administration)) OR (Fluid therapy)) OR (passive leg ras*)) OR (passive leg rasing) | 353606 |
| 7 | #5 AND #6 Filters: Humans | 355 |
|  | | |
| **Web of Science (237 records)** | | |
| **#** | **Details** | **Records** |
| 1 | ((((ALL=(Oxygen saturation)) OR ALL=(Saturation, Oxygen)) OR ALL=(Blood Oxygen Level*)) OR ALL=(Level, Blood Oxygen)) OR ALL=(Oxygen Level, Blood) | 82951 |
| 2 | (((ALL=(Vena Cava, Superior)) OR ALL=(Superior Vena Cavas)) OR ALL=(Superior Vena Cava)) OR ALL=(Central venous*) | 46540 |
| 3 | #1 AND #2 | 1841 |
| 4 | (((((ALL=(ScvO2)) OR ALL=(SvO2)) OR ALL=(Central venous oxygen saturation)) OR ALL=(Central venous blood oxygen saturation)) OR ALL=(Mixed venous oxygen saturation)) OR ALL=(Mixed venous blood oxygen saturation) | 2826 |
| 5 | #3 OR #4 | 3188 |
| 6 | ((((((((ALL=(Fluid responsiveness)) OR ALL=(Volume responsiveness)) OR ALL=(Preload responsiveness)) OR ALL=(Fluid challenge)) OR ALL=(Volume challenge)) OR ALL=(Fluid administration)) OR ALL=(Fluid therapy)) OR ALL=(passive leg rasing)) OR ALL=(passive leg ras*) | 212680 |
| 7 | #5 AND #6 and Article (Document Types) | 237 |
|  | | |
| **EMBASE (518 records)** | | |
| **#** | **Details** | **Records** |
| 1 | 'vena cava, superior'/exp OR 'vena cava, superior' OR (('vena'/exp OR vena) AND cava, AND superior) OR (superior AND vena AND cavas) OR (superior AND vena AND cava) OR (central AND venous*) | 109598 |
| 2 | 'oxygen saturation'/exp OR 'oxygen saturation' OR (('oxygen'/exp OR oxygen) AND saturation) OR (saturation, AND oxygen) OR (level, AND blood AND oxygen) OR (blood AND oxygen AND level*) OR (oxygen AND level, AND blood) | 202883 |
| 3 | #1 AND #2 | 6304 |
| 4 | Scvo2 OR svo2 OR (central AND venous AND oxygen AND saturation) OR (central AND venous AND blood AND oxygen AND saturation) OR (mixed AND venous AND oxygen AND saturation) OR (mixed AND venous AND blood AND oxygen AND saturation) | 6703 |
| 5 | #3 OR #4 | 8654 |
| 6 | 'fluid responsiveness' OR (('fluid'/exp OR fluid) AND responsiveness) OR (volume AND responsiveness) OR (preload AND responsiveness) OR (fluid AND challenge) OR (volume AND challenge) OR (fluid AND administration) OR (fluid AND therapy) OR (passive AND leg AND rasing) OR (passive AND leg AND ras*) | 371724 |
| 7 | #5 AND #6 AND 'human'/de AND 'article'/it | 518 |
|  | | |
| **Cochrane Central Register of Controlled Trials** **(271 records)** | | |
| **#** | **Details** | **Records** |
| 1 | (Vena Cava, Superior):ti,ab,kw OR (Superior Vena Cavas):ti,ab,kw OR (Superior Vena Cava):ti,ab,kw OR (Central venous*):ti,ab,kw | 6458 |
| 2 | (Oxygen saturation):ti,ab,kw OR (Saturation, Oxygen):ti,ab,kw OR (Blood Oxygen Level*):ti,ab,kw OR (Level, Blood Oxygen):ti,ab,kw OR (Oxygen Level, Blood):ti,ab,kw | 23443 |
| 3 | #1 AND #2 | 654 |
| 4 | (ScvO2):ti,ab,kw OR (SvO2):ti,ab,kw OR (Central venous oxygen saturation):ti,ab,kw OR (Central venous blood oxygen saturation):ti,ab,kw | 684 |
| 5 | (Mixed venous oxygen saturation):ti,ab,kw OR (Mixed venous blood oxygen saturation):ti,ab,kw | 298 |
| 6 | #3 OR #4 OR #5 | 1010 |
| 7 | (Fluid responsiveness):ti,ab,kw OR (Volume responsiveness):ti,ab,kw OR (Preload responsiveness):ti,ab,kw OR (Fluid challenge):ti,ab,kw OR (Volume challenge):ti,ab,kw | 26355 |
| 8 | (Fluid administration):ti,ab,kw OR (Fluid therapy):ti,ab,kw OR (passive leg rasing):ti,ab,kw OR (passive leg ras*):ti,ab,kw | 20419 |
| 9 | #7 OR #8 | 42228 |
| 10 | #6 AND #9 | 271 |

| Study No. | Author/year | AUROC | Sensitivity (%) | Specificity (%) | Cutoff value | True positive | False positive | False negative | True negative |
| --- | --- | --- | --- | --- | --- | --- | --- | --- | --- |
| 1 | Giraud/2011 | 0.9 | 86 | 81 | 4% | 12 | 3 | 2 | 13 |
| 2 | Xu/2017 | 0.88 | 78 | 95 | 5% | 14 | 1 | 4 | 21 |
| 3 | Giraud/2021 | 0.89 | 87 | 89 | 4.5% | 13 | 2 | 2 | 16 |
| 4 | Khalil/2021 | 0.84 | 78.7 | 81.5 | 4% | 48 | 5 | 13 | 22 |
| 5 | Nassar/2021 | 0.68 | 64 | 65 | 3.5% | 16 | 8 | 9 | 16 |

## Table S2. Detailed diagnostic accuracy of ScvO_2_ variation for evaluating fluid responsiveness

No. number; ScvO_2_ central venous oxygen saturation; AUROC the area under the receive operator characteristic curve.

# Figure S1. Sensitivity analysis to assess the robustness of ScvO_2_ variation for defining fluid responsiveness by excluding studies introducing a high risk of bias


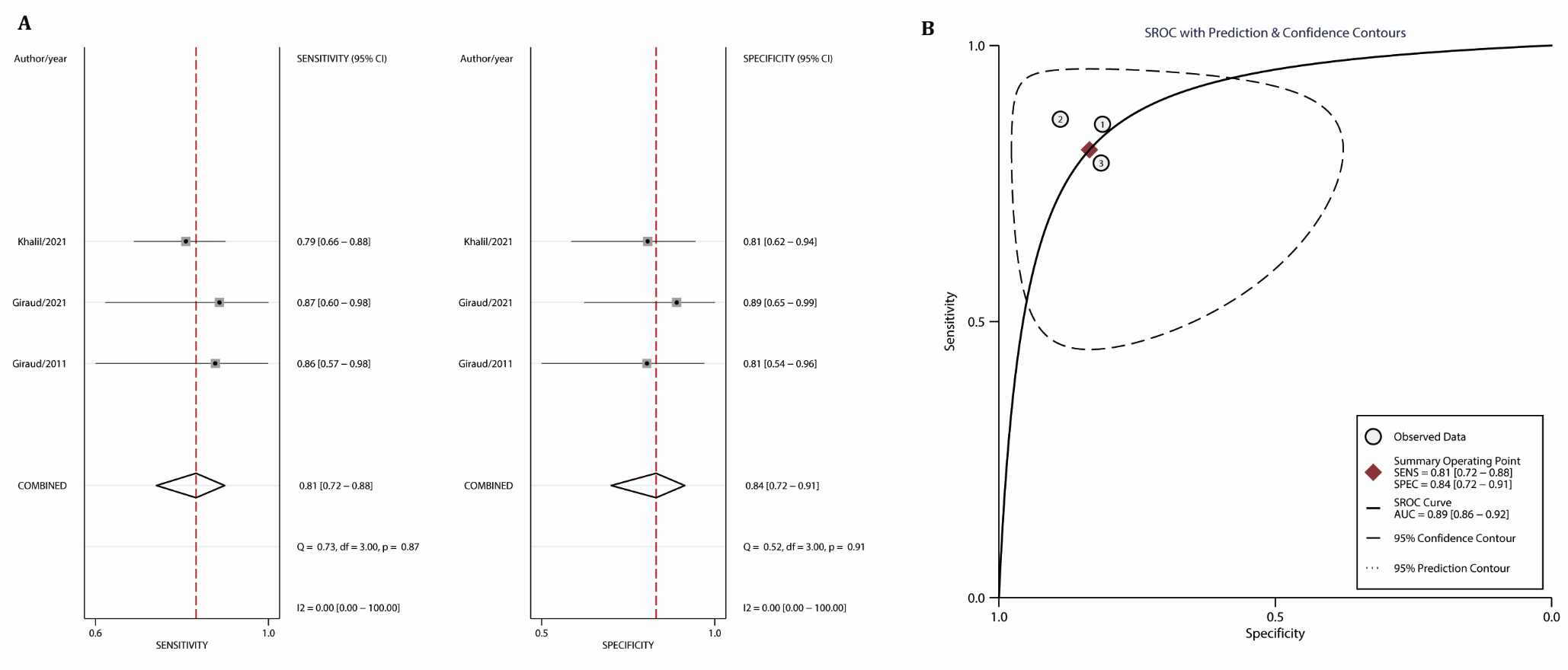


Panel A: Forest plot of the sensitivity and specificity; Panel B: Summary receiver operating characteristic curve. The circle 1, 2, 3 refer to the study by Giraud et al (2011), the study by Giraud et al (2021), and the study by Khalil et al (2021), respectively. The 95% prediction contour cannot be estimated due to the limited included studies in the sensitivity analysis.

SROC summary receiver operating characteristic curve; AUC aera under the curve; SENS sensitivity; SPEC specificity.
